# Experience with Nuclear Medicine Information System

**DOI:** 10.4274/Mirt.29392

**Published:** 2012-12-20

**Authors:** Bilge Volkan-Salanci, Figen Şahin, Vahide Babekoğlu, Ömer Uğur

**Affiliations:** 1 Department of Nuclear Medicine Hacettepe University, Ankara, Turkey; 2 TC Health Bilgi Teknolojileri A.Ş., Ankara, Turkey; 3 Hacettepe University Hospitals, Ankara, Turkey

**Keywords:** Picture archiving and communication systems, hospital information systems, nuclear medicine

## Abstract

**Objective:** Radiology information system (RIS) is basically evolved for the need of radiologists and ignores the vital steps needed for a proper work flow of Nuclear Medicine Department. Moreover, CT/MRI oriented classical PACS systems are far from satisfying Nuclear Physicians like storing dynamic data for reprocessing and quantitative analysis of colored images. Our purpose was to develop a workflow based Nuclear Medicine Information System (NMIS) that fulfills the needs of Nuclear Medicine Department and its integration to hospital PACS system.

**Material and Methods:** Workflow in NMIS uses HL7 (health level seven) and steps include, patient scheduling and retrieving information from HIS (hospital information system), radiopharmacy, acquisition, digital reporting and approval of the reports using Nuclear Medicine specific diagnostic codes. Images and dynamic data from cameras of are sent to and retrieved from PACS system (Corttex©) for reprocessing and quantitative analysis.

**Results:** NMIS has additional functions to the RIS such as radiopharmaceutical management program which includes stock recording of both radioactive and non-radioactive substances, calculation of the radiopharmaceutical dose for individual patient according to body weight and maximum permissible activity, and calculation of radioactivity left per unit volume for each radionuclide according their half lives. Patient scheduling and gamma camera patient work list settings were arranged according to specific Nuclear Medicine procedures. Nuclear Medicine images and reports can be retrieved and viewed from HIS.

**Conclusion: ** NMIS provides functionality to standard RIS and PACS system according to the needs of Nuclear Medicine.

**Conflict of interest:**None declared.

## INTRODUCTION

Traditional paper-based file system is replaced by digital data storage in the last 20 years. Any patient data are processed and stored digitally in hospital information system (HIS), enabling the management of the medical records of the patients, the billing system, administrative and legal aspects of a hospital. HIS enables the classification of patient data, improving the patient management and promotes effective source (i.e. personnel and infrastructure) management (1). HIS requires digital patient data collection which is easier and free of transcription errors. It inhibits duplication of patient entries; therefore data collected is more reliable. And the information can be accessed from any terminal present in the hospital. All these features improve evidence based patient management. When all the hospitals, medical centers and social security organizations establish their own hospital information system it is essential for them to communicate properly which is surmounted by Health level-7 (HL-7) data standards. These provide an interface to share patient information in between different HIS’s. HL7 develops conceptual, documental, application, and messaging standards (2). 

A radiology information system (RIS) is developed for patient data and image management in radiology department and usually integrated with picture archiving and communication system (PACS) (3). RIS system is composed of patient registration, scheduling, work flow management, image collection and reporting capabilities. PACS, on the other hand enables the efficient storage of multimodality digital images, and these stored data can be accessed throughout the hospital. Digital imaging and communications in medicine (DICOM) is generated and used for image storage and transfer in PACS system worldwide. PACS is composed of imaging modalities that acquires patients’ diagnostic images, image storage archieves, intranet for image or data transfer, and workstations for review of the images. It replaces the use of medical films, decreases the cost for image storage, enables the easy and fast retrieving of the digital imaging, therefore provides effective use of picture archive. The DICOM format is compatible with other medical automation systems, and this feature enables to transfer the patient image data not only in the hospital but also in between other medical centers (4). Although nuclear medicine images are acquired and stored in DICOM format, sharing PACS with radiology department sometimes is problematic (5). 

RIS is basically evolved for the need of radiologists and mainly used to schedule patient appointments, to manipulate or to distribute radiographic images/data. However, nuclear medicine is a team work and workflow in a nuclear medicine department is more complicated than a radiology department. Moreover, CT/MRI oriented classical PACS systems for image storage is far from satisfying nuclear physicians. 

A nuclear medicine information system (NMIS) expands the basic functionality of RIS to include additional functions, which are essential for an efficient workflow in nuclear medicine applications like integrated radiopharmaceutical management function. The aim of this study is to develop a workflow based NMIS to fulfill the needs of Nuclear Medicine Department and integration of it to hospital information system and PACS. 

## MATERIALS AND METHODS

We have been using the NMIS which had additional functions added to RIS such as radiopharmaceutical management program. This program includes stock recording of radioactive tracers, drugs and non-radioactive consumables. The program calculates the radiopharmaceutical dose for individual patient according to body weight and maximum permissible activity. Calculation of radioactivity left per unit volume for each radionuclide according to their half lives is also possible using this program. 

Patient scheduling and gamma camera patient work list settings were arranged according to specific Nuclear Medicine procedures. Workflow uses HL7 and steps include patient scheduling and retrieving information from HIS, management of radiopharmaceuticals, radionuclide injection, image acquisition, image transfer, digital reporting and approval of the reports using Nuclear Medicine specific diagnostic codes. Program also enables to summarize the gamma camera or PET camera performance. Static images and dynamic data from cameras of seven different vendors are sent and retrieved from PACS system (Corttex©) for reprocessing and quantitative analysis. Statistics and query according to specific diagnostic codes are possible. Nuclear Medicine images and reports can be retrieved and viewed from hospital intranet by a web based program.

## RESULTS

**Figure 1:**
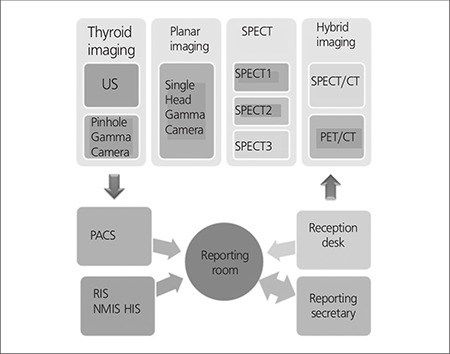
Workflow in nuclear medicine department

The workflow in the nuclear medicine department is as follows (Figure 1). When the referring physician requests a nuclear medicine test for the patient, the request form which includes short clinical information is filled via HIS. This request form is then transferred to nuclear medicine secretary and also appears on secretary workflow in NMIS. The indications for the corresponding procedure are consulted to a nuclear medicine resident or specialist. The appointment is scheduled and the instructions for patient preparation are given to the patient by the nuclear medicine secretary. At the day of the appointment, patient’s entry is activated and imbursement is controlled by the secretary. She then activates the patient workflow in NMIS for the patient’s nuclear medicine procedure. 

In the next step, radiopharmacist sees the related patient information (such as body weight, age, height and procedure) and prepares the desired radiopharmaceutical in the desired dose. The radiopharmacist also controls and drops off the used consumables and radiopharmaceuticals from stock list. This allows tracking of the stock and alerts the radiopharmacist when the stocks are low. The integrated radionuclide management function allows radiopharmacist to record incoming radioactive materials, and keep track of the consumption of radioactive substances. The information such as the radionuclides to be used, their properties and half-lives, the maximum permissible activity in accordance with radioactive exposure regulations and the supplier is also stored in the basic data. 

When the patient dose is ready, the patient information is transferred to nuclear medicine nurse’s workflow. Under the supervision of nuclear medicine resident/specialist the nurse deals with patient preparation (patient hydration, appropriate medication, intravenous line and proper patient position), makes the radiopharmaceutical injection and gives the imaging appointment if it is a static acquisition procedure. However, if a dynamic acquisition is needed, the patient information simultaneously appears on nuclear medicine technologist’s workflow. Then the patient is placed under gamma camera, the i.v. radiopharmaceutical injection and dynamic acquisition is started simultaneously.

For imaging, nuclear medicine technologist consults the acquisition protocol to the nuclear medicine resident/specialist and sets the gamma camera for that specific procedure, prepares the patient for acquisition and starts the acquisition program. 

After the completion of the acquisition, the images are sent to workstation for reporting and to PACS for storage. Any intervention during acquisition such as diuretic injection or any drug administration is also noted and reported to the nuclear medicine resident or specialist. After the completion of the nuclear medicine procedure, the patient information and images are reviewed by the nuclear medicine resident/specialist and findings are simply dictated to reporting secretary via NMIS. The reporting secretary uses the suitable template and writes down the final report in document format. Final document is sent for final approval of nuclear medicine specialist. During final approval, images on PACS system and scintigraphic findings present in nuclear medicine report are reviewed by an experienced nuclear medicine physician and a final diagnosis is set for the patient. After the approval, scintigraphy report appears in hospital intranet and retrieved by patient’s referring physician. 

**Figure 2:**
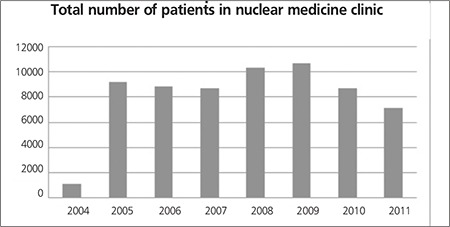
Distribution of Nuclear Medicine procedures according to years,retrieved from NMIS

**Figure 3:**
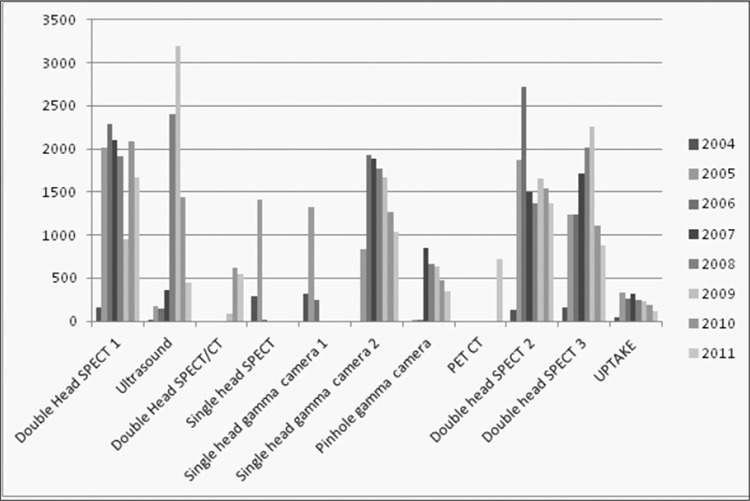
Performance of gamma cameras and other imaging systems

**Figure 4:**
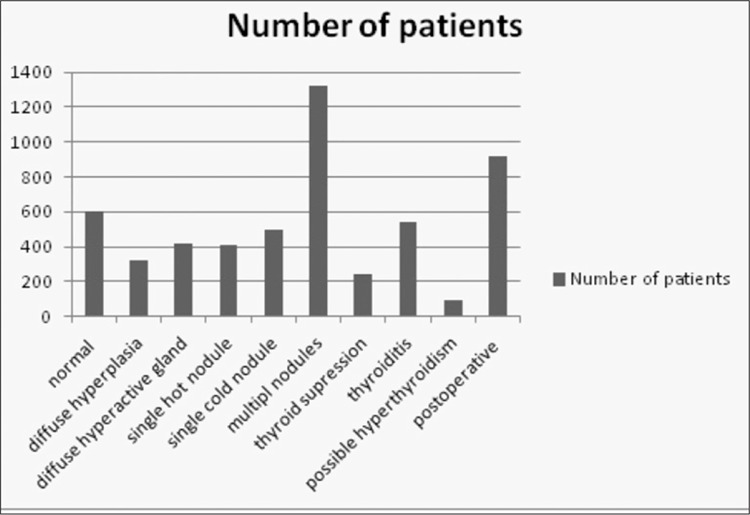
Diagnostic classification for thyroid scintigraphy reports

A total of 62,988 nuclear medicine procedures have been performed, reported using NMIS program and images were restored in PACS, between September 2004 – December 2011 in Hacettepe University nuclear medicine department (Figure 2) when performance statistics was reviewed. An 18% decrease in patient number is observed both in 2010 and 2011 when compared to the previous year. PET/CT examinations form 10% of the imaging protocols performed during 2011. When total number of PET/CT examinations is excluded, it is observed that conventional nuclear medicine procedures decreased by 43%. The performance of cameras and other imaging systems is seen in Figure 3. After acquisition, patient images and clinical data are reported and during this step the report is classified and a proper diagnosis is set by nuclear medicine physician. This diagnostic classification permits patient tracking, prospective or retrospective data collection. Figure 4 shows diagnostic classification for thyroid scintigraphy reports among 7 years. 

**Figure 5:**
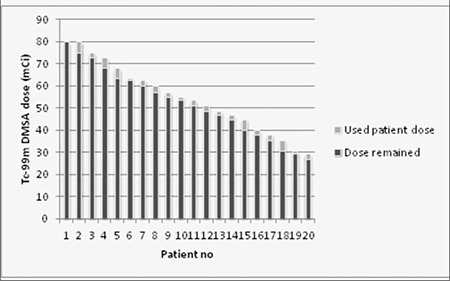
Graph showing remained and used Tc-99m DMSA doses

Radiopharmaceutical preparation and administration is an important feature of NMIS. Table 1 shows the list of radiopharmaceuticals, patient information, doses prepared for patients, and cameras that the acquisitions take place. A total of 11 radionuclides and 17 cold kits have been used for imaging in our routine nuclear medicine clinic during 7 years. Total number of patient appointments and total number of patients are given to the radiopharmacist one day before; the only exception is emergency procedures. However, radiopharmaceutical preparation starts when the patients arrive to the department. The radiopharmacist tracks the patient dose after each injection (Figure 5) and also tracks the cold kits at the end of every week. Some cold kits used in nuclear medicine, such as nanocolloid, have relatively short shelf-life. Therefore their stock management is meticulous. 

**Figure 6:**
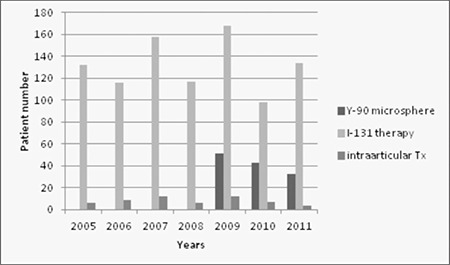
Total number of therapies

Seven different radionuclide therapies are given to patients in our clinic. These are radioiodine therapy for hyperthyroidism, radiosynovectomy, radioiodine therapy for differentiated thyroid cancer, Y-90 microsphere therapy for liver metastasis, I-131 MIBG therapy, radionuclide pain palliation therapy, and radioimmunotherapy for lymphomas. Figure 6 shows the total number of therapies given each year. Radioiodine therapy for hyperthyroidism is given as outpatient and oral solution is used. Therefore, every patient dose is calculated after decay correction. Dose tracking feature of NMIS plays an important role in this therapy. 

## DISCUSSION

NMIS is designed according to HL7 standards and expands the basic functionality of RIS to include additional functions, which are essential for an efficient workflow in nuclear medicine applications. In nuclear medicine, open sources of radioactivity are used and these radioisotopes have either very short or relatively short half-lives that require a strict schedule. Therefore, all the attendees (E.g.: secretary, radiopharmacist, nurse, technician, doctor) should work in harmony. Any delay or problem in this workflow may result in loss of functional data. Images have different formats in nuclear medicine and they should be acquired, stored and retrieved properly in order to retain functional information. NMIS system used in our department fulfills these requirements. A delay in any step is quickly realized and corrected. NMIS helps to manage all the personnel, cameras and radioactive stock more efficiently. Moreover, it reveals a reliable data set for both retrospective and prospective clinical studies.

Using NMIS, the status of every process is known and tracked at any time to maintain workflow management. This includes transfer of patient data from HIS to NMIS, patient scheduling, radiopharmaceutical preparation, radioactive decay and stock management, transfer of work list to gamma cameras, and DICOM based work flow within the department. NMIS retrieves patient information from HIS when the nuclear medicine secretary completes the registration. This connection prohibits repetitive entries and allows matching the clinical problem and nuclear medicine procedure which also forms a control step for reimbursement issues. The secretary also confirms the social security and the payment of each patient. The contact information is verified and any change is entered to both NMIS and HIS. The essential patient information such as body weight, age etc. is also checked by nuclear medicine secretary and this data is transferred to both radiopharmacist and nurse workflow. 

Nuclear medicine procedures are tightly scheduled. Nuclear medicine protocols require prolonged follow-up of the patients and some studies span several days. Therefore, patient scheduling is quite different from regular radiology appointments (4). Some centers, especially the imaging departments overcome this problem by integrating NMIS and RIS and a specified order system (4). However, in our institution radiology and nuclear medicine departments work independently and NMIS provides a good solution to this problem. Correct dosing of radionuclide before administration is another issue and knowledge of this data is essential during interpretation. Proper timing of radiotracer or drug injections such as diuretics is a necessity when obtaining and interpreting functional data. This step is successfully covered by NMIS. All the patient information is available in NMIS and the work flow is tightly scheduled. Therefore NMIS software not only decreases the time for patient preparation but also decreases the possibility of medication errors such as injection of wrong radiopharmaceutical to wrong patient in wrong dose.

There are some efforts to integrate HIS with RIS and PACS, by designing advanced RIS (6,7). Such software not only establishes a transfer system between RIS and HIS but also in between the different structural units of RIS. Moreover it manages the translation between HL7 and DICOM (6). In our institution HIS and PACS are still running independently. Although the workflow is managed by NMIS, the acquired images (i.e. raw data and processed images in JPEG format) are sent to PACS or retrieved from PACS. The digital medical reporting and approval is possible in NMIS and this step is in connection with HIS enabling distribution of digital reports with radiology and/or other departments. The processed images are also shared with the whole hospital however reprocessing of raw data is only possible in nuclear medicine terminals. 

There are different acquisition schemes in nuclear medicine. Images obtained using different tracers are acquired in different energy windows, even in different camera systems. Image resolution depends on the radiotracer, camera properties and imaging protocols. The dynamic acquisitions contain multiple frames that should be stored and retrieved for reprocessing. Sometimes nuclear medicine cameras have two, even three heads and images obtained from multiple heads should be stored and re-united properly. Any loss in image acquisition information (E.g.: detector number, acquisition angle etc.) results with the loss of that functional data and prevents reconstruction. Images in nuclear medicine include functional data hence quantitative/semi-quantitative analysis should be carried out using colored images. These functional images are essential for patient follow-up. Therefore, converting images to static image formats as done in radiology may be problematic in nuclear medicine (8). Whole-body hybrid imaging contains sectional images of both functional, such as PET or SPECT, and anatomical data, such as CT or MRI. These image sets contain high amount of data which can be a burden to PACS systems. The images in nuclear medicine should be acquired and stored in DICOM format without loss of functional data as done in NMIS. There are some efforts to develop interfaces that allow navigating between multimodality image sets (9). In our institution same storage system is used both by nuclear medicine and radiology departments. The raw data of all nuclear medicine acquisitions are stored in DICOM format in PACS and retrieved without loss of any functional data such as injected dose of radiopharmaceutical, such as F-18 FDG, injection time of the radiopharmaceutical patient age, gender or body weight etc.) from nuclear medicine work terminals. However, the snapshots derived from reformatted images (SPECT slices, esp. PET/CT fused images) can be viewed by referring physician via intranet. 

Nuclear medicine depends on consumables and radiopharmaceuticals; therefore tracking of stock list daily, monthly and annually is meticulous. There is intensive turnover of different radiotracers, pharmaceuticals and other consumables and their management is a major problem in nuclear medicine. NMIS used in our institution both provides management of stock of consumables and daily monitoring of radiopharmaceuticals (Figure 5). This allows utilizing every radiopharmaceutical and making proper appointments. Furthermore the software allows the tracking and correction of billing errors. 

The performance report feature of NMIS is a useful tool for following the current trends in hospital. The information derived from this database can help to develop new management strategies. Camera performance reports reveal camera performance (malfunctioning) and this information not only helps to organize personnel work plan but also provides to foresee future investments. The statistical results retrieved from NMIS archieve system revealed that the total number of patients decreased gradually during last 3 years (Figure 2) however more complicated procedures –such as SPECT, SPECT/CT or PET/CT- were carried out during this time period (Figure 3). The radionuclide therapies were also increased during the last three years which shows a change in general trends and in patient population. Furthermore, it is possible to retrieve performance reports and nuclear medicine specific diagnostic codes for research purposes. The patient data can be derived from both NMIS and HIS, ensuring the accuracy, and increases the reliability of retrospective data. The statistics derived from NMIS also provides fast and easy planning of prospective research. 

One limitation of NMIS software is that some procedures especially ones acquired by older camera systems lose their private elements when retrieved from PACS. Nuclear medicine raw images can be reprocessed by DICOM stations. This is quite advantageous because functional data and raw images are for private use of nuclear medicine, however images in picture format is displayed for common workstations of HIS. Further effort should be spent for integrating NMIS with PACS and HIS, providing a more user friendly version and integrating DICOM and PACS for better handling of functional images. 

NMIS software was mainly designed for the management of patient information and radiopharmaceuticals in a nuclear medicine department. It does not include any features for the management of camera or dose calibrator quality control, radioactive waste control, contamination monitoring, or personal dose monitoring which are also essential for a nuclear medicine department. Updates of NMIS is planned to cover these topics in the future.

## CONCLUSION

Nuclear medicine depends on radionuclides, pharmaceutical kits and other consumables and their stock management both in short-term and long-term is meticulous. Images acquired in nuclear medicine contain functional data, which have to be handled and stored in a different manner when compared to radiology. NMIS software designed for management of routine workflow in nuclear medicine provides functionality to standard RIS and PACS system.

**Acknowledgement**

The authours would like to thank Nida B. Kesimli, Diren Düzgünoğlu, Aydın Bakır, Soner Birinci and Haluk Çelikel for their support.

## Figures and Tables

**Table 1:**
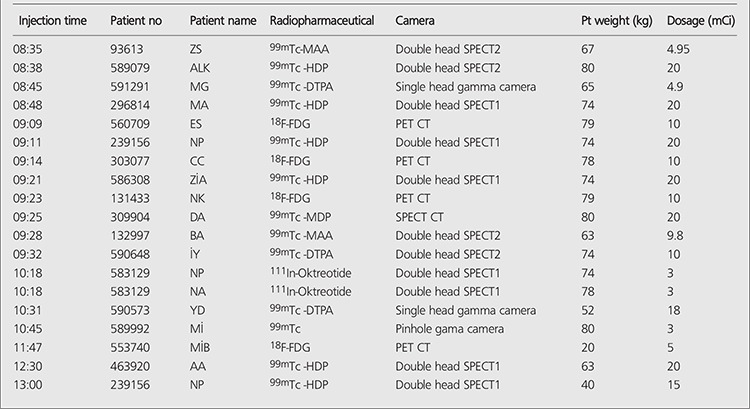
Daily patient information obtained from NMIS
